# Osteoporosis increases subsequent risk of gallstone: a nationwide population-based cohort study in Taiwan

**DOI:** 10.1186/s12876-014-0192-z

**Published:** 2014-11-18

**Authors:** Sukhontip Klahan, Chun-Nan Kuo, Shu-Chen Chien, Yea-Wen Lin, Chun-Yi Lin, Chia-Hsien Lin, Wei-Chiao Chang, Ching-I Lin, Kuo-Sheng Hung, Wei-Pin Chang

**Affiliations:** Department of Clinical Pharmacy, School of Pharmacy, Taipei Medical University, Taipei, Taiwan; Department of Pharmacy, Taipei Medical University-Wan Fang Hospital, Taipei, Taiwan; Department of Pharmacy, Taipei Medical University Hospital, Taipei, Taiwan; Department of Healthcare Management, Yuanpei University of Medical Technology, HsinChu, Taiwan; Department of Health Industry Management, Kainan University, Taoyuan, Taiwan; Department of Neurosurgery, Clinical Research Center, Graduate Institute of Injury Prevention and Control, Taipei Medical University, Wan Fang Hospital, Taipei, Taiwan; Master Program for Clinical Pharmacogenomics and Pharmacoproteomics, School of Pharmacy, Taipei Medical University, Taipei, Taiwan; Graduate Institute of Pharmacognosy, Taipei Medical University, Taipei, Taiwan; Department of Nutrition and Health Sciences, Kainan University, Taoyuan, Taiwan; Comprehensive Cancer Center of Taipei Medical University, Taipei, Taiwan

**Keywords:** Osteoporosis, Gallstone, Population-based study, Taiwan

## Abstract

**Background:**

Osteopontin (OPN) is a pro-inflammatory cytokine which is expressed in various tissues. It participates in the bone remodeling process and stimulates bone resorption by osteoclasts. It is also a core protein of cholesterol gallstones. We hypothesized osteoporotic patients might have higher risk in developing gallstones and conducted a population-based study to examine the risk of developing gallstone in osteoporotic patients in Taiwan.

**Methods:**

A total of 1,638 patients diagnosed with osteoporosis between 2003 and 2005 were identified in the National Health Insurance Research Database. A comparison cohort without osteoporosis (*n* =6,552) was randomly matched to each osteoporosis patient at a ratio of 4: 1 based on age and sex. A Cox proportional-hazards regression analysis was performed to evaluate the 5-year gallstone-free survival rates for the 2 cohorts.

**Results:**

During the 5-year follow-up period, 114 and 311 cases of gallstone occurred in the osteoporosis and comparison cohorts, respectively. After adjusting for the confounders, the Cox regression analysis of the risk of gallstone in the osteoporosis and comparison cohorts yielded a hazard ratio of 1.35 (95% confidence interval: 1.07 - 1.69; *p* < 0 .01).

**Conclusion:**

Patients with osteoporosis in Taiwan have a higher risk of developing gallstone than the general population.

## Background

Both osteoporosis and gallstone are diseases that affect elderly patients worldwide. Osteoporosis is a chronic, multifactorial, systemic skeletal disease characterized by low bone density and an increased risk of bone fractures [1]. Bone fracture is a major consequence of osteoporosis. Although osteoporosis was previously thought to be associated with aging, the causes of osteoporosis are related to the effects of inflammatory mediators and endogenous hormones, such as estrogen deficiency [2]. Estrogen deficiency is associated with bone resorption caused by increased numbers of osteoclasts and increased their activity [3]. Recent studies have also demonstrated that pro-inflammatory cytokines, such as interleukin (IL)-1, tumor necrosis factor-α, and IL-6, are primary mediators of accelerated bone loss following menopause [4]. In addition, the up-regulation of T lymphocytes are crucial in the stimulating osteoclasts in post-menopausal bone loss [5].

Biliary calculus, also known as gallstone, is a crystalline solid formed from bile components that occurs primarily in the gallbladder. Gallstone is a common and costly disease and its complications consume about $6.5 billion in the United States [6]. Gallstones are classified as cholesterol, pigment, or mixed stones [7] based on whether the stones consist primarily of cholesterol, bilirubin, or calcium deposits. Environmental and genetic factors, including female sex [8,9], family medical history [10], age [11-14], and estrogen-replacement therapy [15], contribute to the development of gallstone.

Osteopontin (OPN) is a pro-inflammatory cytokine that is expressed in various tissues involved in a wide range of biological processes, such as bone mineralization, inflammation, and cell survival [16]. It is expressed at high levels in the bone matrix, and may promote the survival of autoreactive T cells [17] and participates in the bone remodeling process [18]. One study showed that it stimulates the adhesion, migration and bone resorption by osteoclasts [19]. Fodor et al. found that high levels of OPN in postmenopausal women are associated with low bone mineral density, increased levels of bone turnover markers, and osteoporotic vertebral fractures [20]. A recent study has shown that OPN is also a core protein in the formation of cholesterol gallstone [21]. Based on the studies mentioned above, we hypothesized that osteoporotic patients might have higher risk in developing gallstones, but the association is not investigated. In the present study, we intended to examine the relationship between osteoporosis and the risk of developing gallstones in Taiwan using a nationwide, population-based dataset.

## Methods

### Data source

The National Health Insurance (NHI) program was implemented in 1995, and provides reimbursements for health-care costs to 99% of the 23 million residents of Taiwan. Claims data are maintained in the National Health Insurance Research Database (NHIRD), which is managed by the National Health Research Institutes (NHRI). Our study used the Longitudinal Health Insurance Database (LHID2005), which is a subset of the NHIRD. The LHID2005 database allows researchers to follow-up all the medical service utilization for these 1 million enrollees that randomly selected from the NHIRD in 2005. The NHRI claimed that there are no statistically significant differences related to age, sex, or health-care costs between the LHID2005 and the NHIRD.

We examined the ambulatory and inpatient care data for patients in the LHID2005 from 1997 to 2010. The original identification number of each patient in the LHID2005 is encrypted to maintain patient privacy, and the encrypted numbers are linked to the health-care data for each patient. This study was exempt from full review by joint institutional review board of Taipei Medical University (TMU-JIRB No.201306039) because the LHID2005 consist of de-identified secondary data released to the public for research purposes.

### Participants

We used a study cohort and a comparison cohort to retrospectively examine the relationship between osteoporosis and gallstone. We identified patients aged 50 years or older who were newly diagnosed with osteoporosis between January 1, 2003, and December 31, 2005, based on the diagnostic criteria of the *International Classification of Diseases, Ninth Revision, Clinical Modification* (*ICD-9*-*CM* 733.X). The date of the first osteoporosis diagnosis for each patient was assigned as their index date for our study. To ensure the accuracy of the data, only patients with ≥ 2 ambulatory visits or with ≥ 1 inpatient visit for osteoporosis and receiving at least one bone mineral density examination were included in the osteoporotic cohort. Four patients without osteoporosis were randomly matched based on age, sex, and the index year to each patient in the osteoporosis cohort. Each subject was tracked for five years from their index date to identify whether they had suffered from a gallstone. The definition of the gallstone cases in this study was that if they received ≥ 2 gallstone diagnoses for ambulatory care visit or ≥1 diagnosis for inpatient care, and received at least one abdominal ultrasound examination. Patients with a history of gallstone (*ICD-9*-*CM* 574.X) were excluded from our study. Hypertension (*ICD-9*-*CM* 401.X-405.X), diabetes mellitus (*ICD-9*-*CM* 250.X), hyperlipidemia (*ICD-9*-*CM* 272.X), obesity (*ICD-9*-*CM* 278.X), liver cirrhosis (*ICD-9*-*CM* 571.X), hemolytic anemia (*ICD-9*-*CM* 282.X-283.X), spinal cord injury (*ICD-9*-*CM* 806.X, 907.2, 952.X), receiving hormone replacement therapy or clofibrate or total parenteral nutrition over 30 days, receiving ceftriaxone over 7 days, receiving short-acting octreotide over 14 days and receiving long-acting octreotide over 3 months were treated as covariates in our analysis of the risk of gallstone.

### Levels of urbanization

The 359 communities in Taiwan were stratified into 8 urbanization categories in the LHID 2005 according to the criteria established by the NHRI, with 1 indicating the most urbanized and 8 indicating the least urbanized. The criteria included population density (persons per km^2^), the percentage of people with a college-level education or higher, the percentage of people aged 65 years or older, the percentage of agricultural workers in the local population, and the number of physicians per 100 000 population. However, because the number of osteoporosis cases in levels 5, 6, 7, and 8 were low, these levels were combined into a single urbanization group, level 5.

### Statistical analysis

The parametric continuous data for the 2 cohorts was compared using a Students *t* test, and the categorical variables were evaluated using the chi-squared test. Gallstone-free survival was calculated for all patients diagnosed with osteoporosis from the date of the first hospitalization or ambulatory visit for gallstone and the end of the study period (December 31, 2010) or death, whichever came first. A Cox proportional-hazards regression analysis stratified by sex, age group, and index year was performed to examine the risk of gallstone in the osteoporosis and comparison cohorts during the 5-year follow-up period. We also examined the effect of sex, age, hypertension, diabetes, and hyperlipidemia on the association between osteoporosis and gallstone events. For the stratified Cox regression analysis, patients were divided into 3 categories, 50 to 64, 65 to 79, or ≥ 80 years. The hazard ratios (HRs) and 95% confidence intervals (CIs) were calculated to represent the risk of gallstone in the cohorts before and after stratification based on age or sex. We computed all study data with Statistical Package for Social Science software version 18 for Windows (SPSS Inc., Chicago, Illinois, USA). The differences between compared groups were considered significant if 2-side p-values were smaller than 0.05.

## Results

A total of 1,638 and 6,552 patients were included in the osteoporosis and comparison cohorts, respectively. The results of our analysis of the sociodemographic and comorbidity data for the osteoporosis and comparison cohorts are shown in Table 1. The osteoporotic patients had higher rates of hypertension, hyperlipidemia, diabetes, spinal cord injury, liver cirrhosis, receiving hormone replacement therapy or clofibrate over 30 days than the comparison cohort, and were more likely to have a moderate monthly income or reside in central or eastern Taiwan.

**Table 1:** **Demographic characteristics and comorbidities for the osteoporosis and comparison cohorts from 2003 to 2005 (** ***N*** **=8190)**

	**Patients with osteoporosis (** ***n*** **=1638)**		**Patients without osteoporosis (** ***n*** **=6552)**		***P*** **value**
	***n***	**%**	***n***	**%**	
**Sex**					1
Male	242	14.8	968	14.8	
Female	1396	85.2	5584	85.2	
**Age (y)**					1
50–64	648	39.6	2592	39.6	
65–79	808	49.3	3232	49.3	
≥ 80	182	11.1	728	11.1	
**Mean ± SD of follow-up (y)**					< .001
	4.76	0.97	4.88	0.66	
**Urbanization level**					0.037
1 (most urbanized)	443	27.0	2010	30.7	
2	452	27.6	1688	25.8	
3	229	14.0	929	14.2	
4	264	16.1	1035	15.8	
5 (least urbanized)	250	15.3	890	13.6	
**Monthly income**					0.006
0	567	34.6	2520	38.5	
NT$ 1–15,840	245	15.0	809	12.3	
NT$ 15,841–25,000	695	42.4	2724	41.6	
NT$ ≥25,001	131	8.0	499	7.6	
**Geographic region**					< .001
Northern	687	41.9	2953	45.1	
Central	467	28.5	1630	24.9	
Southern	355	21.7	1566	23.9	
Eastern	129	7.9	403	6.2	
**Hypertension**					< .001
Yes	1212	74.0	4548	69.4	
No	426	26.0	2004	30.6	
**Hyperlipidemia**					< .001
Yes	895	54.6	2914	44.5	
No	743	45.4	3638	55.5	
**Diabetes**					0.008
Yes	658	40.2	2399	36.6	
No	980	59.8	4153	63.4	
**Liver cirrhosis**					< .001
Yes	368	22.5	900	13.7	
No	1270	77.5	5652	86.3	
**Hemolytic anemia**					0.773
Yes	2	0.1	10	0.2	
No	1636	99.9	6542	99.8	
**Spinal cord injury**					<.001
Yes	99	6.0	17	0.3	
No	1539	94.0	6535	99.7	
**Obesity**					0.826
Yes	10	0.6	37	0.6	
No	1628	99.4	6515	99.4	
**Hormone replacement**					<.001
Yes	231	14.1	298	4.5	
No	1407	85.9	6254	95.5	
**Ceftriaxone**					0.813
Yes	4	0.2	14	0.2	
No	1634	99.8	6538	99.8	
**Clofibrate**					0.045
Yes	1	0.1	Na	Na	
No	1637	99.9	6552	100.0	
**Octreotide**					0.724
Yes	2	0.1	6	0.1	
No	1636	99.9	6546	99.9	
**Total parenteral nutrition**					0.070
Yes	1	0.1	21	0.3	
No	1637	99.9	6531	99.7	

**Figure 1:**
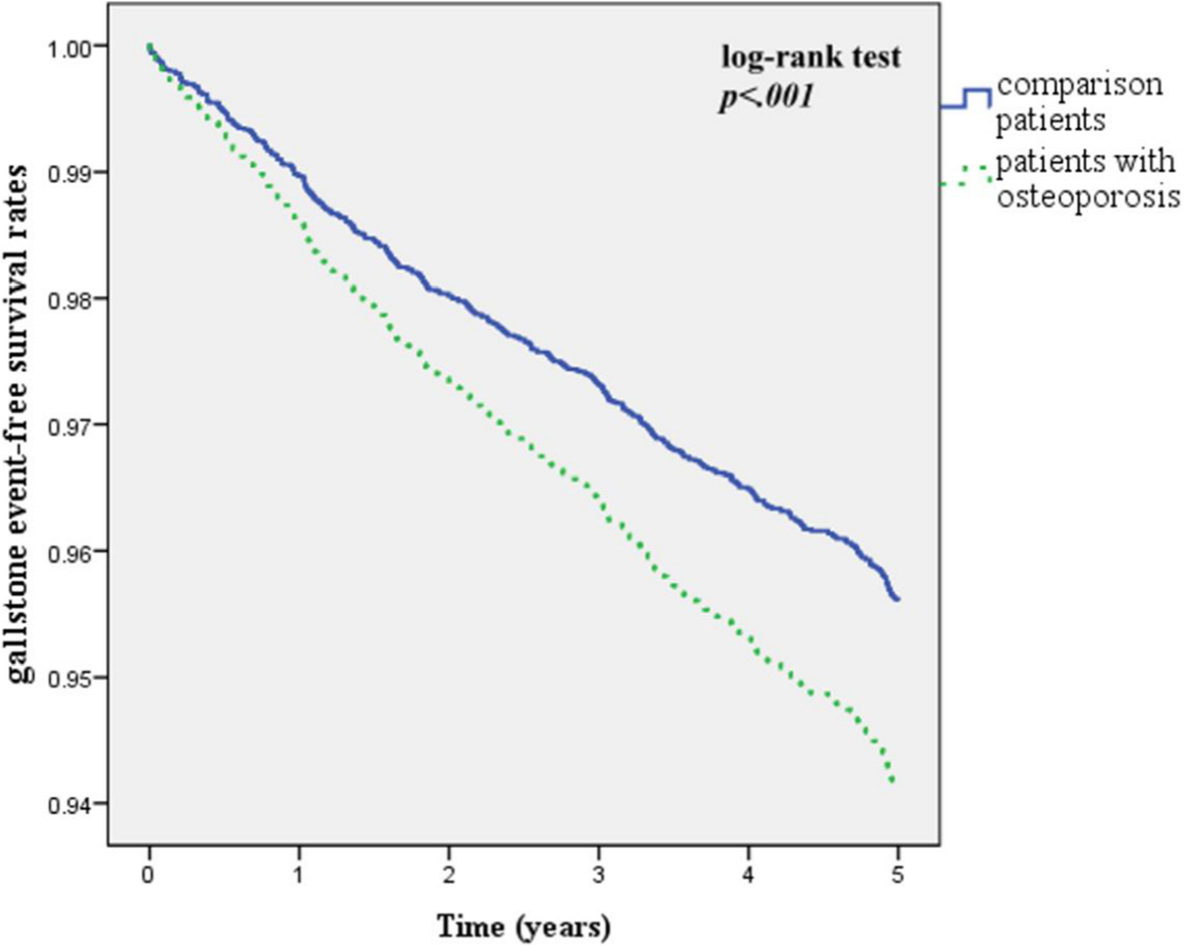
**Gallstone event-free survival rates for subjects with osteoporosis and the comparison group from 2003 to 2005.**

During the 5-year follow-up period, 114 (7.0%) of the osteoporotic patients and 311 (4.7%) of the comparison patients developed gallstones. The Cox regression analysis showed that the crude HR of gallstone was 1.50 times greater (95% CI: 1.21–1.86) for the osteoporosis patients than that of the comparison cohort. The risk of gallstone remained significant after adjusting for potential confounders (adjusted HR: 1.35, 95% CI: 1.07–1.69; Table 2), and the osteoporosis patients had a significantly lower 5-year gallstone-free survival rate (*p* < 0 .001; Figure 1).

**Table 2:** **Incidence of gallstone among the osteoporosis and comparison patients from 2003 to 2005 (** ***N*** **=8190)**

	**Total**		**Patients with osteoporosis**		**Patients without osteoporosis**	
**Gallstone cases**	***n***	**%**	***n***	**%**	***n***	**%**
5-year follow-up period						
Yes	425	5.2	114	7.0	311	4.7
No	7765	94.8	1524	93.0	6241	95.3
Crude HR (95% CI)				1.50 (1.21 - 1.86)*		1
Adjusted HR (95% CI)				1.35 (1.07 - 1.69)**		1

*p <0.001, **p <0.05.

Both crude and adjusted hazard ratios (HRs) and 95% confidence intervals (CIs) were calculated using Cox proportional-hazard regressions stratified by age and sex.

Adjustments are made for sex, age, patients’ monthly income, region, urbanization level, hypertension, hyperlipidemia, diabetes, cirrhosis, spinal cord injury, hormone replacement, and clofibrate.

The overall gallstone incidence density was higher among the patients with osteoporosis (14.62 per 1,000 person-years) than in the comparison cohort (9.73 per 1,000 person-years). The stratified analysis of incidence showed that men had a higher incidence of gallstone than women, and that patients aged 65 to 79 years had the highest incidence of gallstone, compared with the patients ≤ 64 or ≥ 80 years of age in both groups (Table 3).

**Table 3:** **Overall and age- and sex-specific incidence densities and relative hazard of gallstone in the osteoporosis and comparison cohorts**

**Factor**	**Osteoporotic group**				**Comparative group**				**Crude HR**	**Adjust HR**
	**Incident cases**	**Person-years**	**ID** ^**1**^	**95% CI**	**Incident cases**	**Person-years**	**ID** ^**1**^	**95% CI**		
Age (y)^a^										
50-64	42	3080.05	13.64	9.54 - 17.73	103	12702.40	8.11	6.55 - 9.67	1.68 (1.17 - 2.40)**	1.56 (1.06 - 2.28)*
65-79	60	3846.65	15.60	11.68 - 19.51	170	15720.01	10.81	9.20 - 12.43	1.44 (1.07 - 1.94)*	1.25 (0.92 - 1.71)
≥ 80	12	868.91	13.81	6.05 - 21.57	38	3544.48	10.72	7.33 - 14.11	1.28 (0.67 - 2.46)	1.31 (0.64 - 2.68)
Sex^b^										
Men	17	1142.92	14.87	7.86 - 21.89	48	4719.56	10.17	7.31 - 13.03	1.46 (0.84 - 2.54)	1.09 (0.60 - 1.96)
Women	97	6652.69	14.58	11.70 - 17.46	263	27247.33	9.65	8.49 - 10.81	1.51 (1.20 - 1.91)**	1.40 (1.09 - 1.79)**
Total	114	7795.61	14.62	11.96 - 17.29	311	31966.89	9.73	8.65 - 10.80	1.50 (1.21 - 1.86)***	1.35 (1.07 - 1.69)*

*p <0.05,**p <0.01, ***p <0.001.

HR, hazard ratio; ID, incidence density (per 1000 patient-years); CI, confidence interval.

^1^Based on the Poisson assumption.

^a^Adjusted for sex, age, monthly income, region, urbanization level, hypertension, hyperlipidemia, diabetes, cirrhosis, spinal cord injury, hormone replacement, and clofibrate.

^b^Adjusted for age, monthly income, region, urbanization level, hypertension, hyperlipidemia, diabetes, cirrhosis, spinal cord injury, hormone replacement, and clofibrate.

The stratified Cox regression analysis showed that women with osteoporosis had a higher risk of gallstone than those without osteoporosis (adjusted HR: 1.40, 95% CI: 1.09 – 1.79, *p* < 0.01) (Table 3), and osteoporotic patients who aged 50 to 64 years had higher risk of gallstone than non-osteoporotic subjects (adjusted HR: 1.56, 95% CI: 1.06 – 2.28, *p* < 0.01). No significant increased risk was observed for patients aged 65- to 79-year-old or above 80-year-old in either the osteoporosis or comparison cohorts.

## Discussion

Our study is the first to demonstrate a relationship between osteoporosis and gallstone. Nearly 2,000 osteoporotic patients in Taiwan were collected in our current study and compared with non-osteoporotic subjects. As shown in Table 2, our results indicated that osteoporosis was significantly associated with the risk of gallstone.

Osteoporosis and gallstone disease share similar epidemiological characteristic. Female sex is a risk factor for both osteoporosis and gallstones. Sex hormones might play an important role in the development of both osteoporosis and gallstone. D’Amelio et al. showed that estrogen deprivation induces bone loss by up-regulating osteoclastogenesis, which contributes to the development of osteoporosis [5]. Estrogen can promote the hepatic secretion of biliary cholesterol that induces an increase in cholesterol saturation of bile and then increases the risk for the formation of cholesterol gallstones [22]. Our study showed women were more among osteoporotic patients. Women also had significantly higher incidence developing gallstones and women with osteoporosis had a greater risk of gallstones than women without osteoporosis. In addition, although estrogen replacement is a common treatment for osteoporosis in postmenopausal women, studies have suggested that estrogen replacement therapy may be a risk factor for gallstone [23]. Thus, estrogen replacement therapy might affect the risk of gallstone in women with osteoporosis. After adjusting the confounding factor, our result showed that osteoporotic patients still had significantly higher risk in developing gallstones.

Age is another risk factor for both osteoporosis and gallstones. Osteoporosis is highly prevalent, especially in postmenopausal women. Approximately 46% of women will experience at least one osteoporotic fracture after the age of 50 years [24]. In Sirmione study, the incidence of gallstones between the ages of 40–69 years was four times higher than that in younger subjects [25]. The population in our study was at higher risk to develop gallstones because we included those age over 50 years old. We used age-matched control group for following-up. Hence, age would not be a confounding factor in our study.

Other risk factors for the formation of gallstones include family history, obesity, diabetes mellitus, hyperlipidemia, liver cirrhosis, drugs, decreased physical activity, spinal cord injury and hemolysis anemia. After adjusting these confounding factors except family history and decreased physical activity, patients with osteoporosis remained having significantly higher risk in developing gallstones than those without osteoporosis. Because our study was based on NHIRD, family history and physical activity cannot be retrieved from this dataset and not all obesity subjects had a diagnosis of obesity. These would be a limitation in our study. Despite of this limitation, we eliminated the interference of most possible risk factors for gallstones, and the result based on the large sample size showed osteoporotic patients are at higher risk to develop gallstones. Since the complication of gallstones may cause high costs, more effort should be invested in the population to reduce such related cost.

Chronic inflammation might play a key role in the development of gallstone. OPN is a pro-inflammatory cytokine that is expressed in various tissues involved in a wide range of biological processes [16]. OPN participates in the bone remodeling process [18] and stimulates the adhesion, migration and bone resorption by osteoclasts [19]. OPN is also the core protein in cholesterol gallstones [26]. The OPN molecule binds hydroxyapatite and calcium ions, inhibiting the nucleation of cholesterol crystals in vivo. Yang et al. showed that the OPN-mediated inhibition of cholesterol stone nucleation is dose-dependent in an animal model and in vitro using bile from human gallbladders [27]. In a mouse model of gallstone formation, the level of OPN in the gallbladder wall increased before the induction of inflammation [21]. Our results are consistent with the findings of these previous studies, and confirm the correlation between osteoporosis and gallstone disease. Future studies are warranted to identify the role of OPN in the formation of gallstones in patients with osteoporosis.

Certain limitations to our study should be considered. First, the NHIRD files did not provide information regarding family history, physical activity and dietary habits, all of which might be risk factors for gallstone. Second, data regarding serum levels of osteopontin, sex hormones, inflammatory mediators, and blood calcium were also lacking. Third, not all over-weight subjects would have a diagnosis of obesity in NHIRD. Obesity is known to increase the risk of gallstone. Thus, we could not completely exclude the interference of the risk factor.

In our current study, we included much comorbidity as risk factors in our analysis, and adjusted our Cox regression model for them to avoid bias related to the demographic characteristics of the osteoporosis patients. Our study is the first nationwide population-based cohort study with a large sample size to show that osteoporosis is significantly associated with an increased risk for gallstone in Taiwan. Our findings may provide important information to aid medical care plans in the monitoring and prevention of gallstone disease in osteoporosis patients. But the biological mechanism underlying the contribution of osteoporosis to gallstone remains unclear. Future studies of gallstone in osteoporosis patients are needed to confirm our findings.

## Conclusions

This study indicated that osteoporosis patients are at a higher risk of developing gallstone than the general population.
